# The Basics of Electronic Structure Theory for Periodic Systems

**DOI:** 10.3389/fchem.2019.00106

**Published:** 2019-03-13

**Authors:** Peter Kratzer, Jörg Neugebauer

**Affiliations:** ^1^Faculty of Physics, University of Duisburg-Essen, Duisburg, Germany; ^2^Max-Planck-Institut für Eisenforschung, Düsseldorf, Germany

**Keywords:** density functional theory, high-throughput calculations, Brillouin zone sampling, supercell approach, convergence tests, solid-state chemistry techniques

## Abstract

When density functional theory is used to describe the electronic structure of periodic systems, the application of Bloch's theorem to the Kohn-Sham wavefunctions greatly facilitates the calculations. In this paper of the series, the concepts needed to model infinite systems are introduced. These comprise the unit cell in real space, as well as its counterpart in reciprocal space, the Brillouin zone. Grids for sampling the Brillouin zone and finite k-point sets are discussed. For metallic systems, these tools need to be complemented by methods to determine the Fermi energy and the Fermi surface. Various schemes for broadening the distribution function around the Fermi energy are presented and the approximations involved are discussed. In order to obtain an interpretation of electronic structure calculations in terms of physics, the concepts of bandstructures and atom-projected and/or orbital-projected density of states are useful. Aspects of convergence with the number of basis functions and the number of k-points need to be addressed specifically for each physical property. The importance of this issue will be exemplified for force constant calculations and simulations of finite-temperature properties of materials. The methods developed for periodic systems carry over, with some reservations, to less symmetric situations by working with a supercell. The chapter closes with an outlook to the use of supercell calculations for surfaces and interfaces of crystals.

## 1. Introduction

Three-dimensional periodic solids were among the first systems for which the theory of electronic structure was worked out. The concept of an electronic band structure reaches back to the first decade after the invention of quantum mechanics and early examples can be found in the work of Sommerfeld and Bethe (1933), Slater (1934) and others. Almost fifty years ago, the total energy of the electrons and nuclei in an elementary unit cell of a crystalline solid has come into focus of theoretical investigations. To obtain it, summation over reciprocal space (or **k**-space, as it's often called) is required. Thus, through the 1970s, a number of papers about the sampling of **k**-space have been published (Jepsen and Andersen, 1971; Baldereschi, 1973; Chadi and Cohen, 1973; Cunningham, 1974; Monkhorst and Pack, 1976). Given that computational power in these days was rather limited, the emphasis at this time was to keep the number of **k**-points to be treated as small as possible, and the most efficient choice of special **k**-points was the major topic of these earlier works.

Meanwhile, this knowledge has found its way into textbooks. The interested reader may e.g., consult chapter 4 in Martin (2004). With the increase of computer power, computational physicists and materials scientists started to work on more and more complex systems comprising hundreds of atoms in one unit cell. Since such large unit cell goes along with small Brillouin zone (BZ) in reciprocal space, sampling of **k**-space received less attention. Moreover, liquid or amorphous systems that lack translational order were approximated by large supercells, e.g., using quasi-random structures. Due to the lack of 'true' physical periodicity, calculations with large supercells often employ just one **k**-point which, for the sake of additional computational savings, is often chosen to be the Γ-point, i.e., the origin in reciprocal space. As becomes apparent from the bibliography of this review article, only few papers about **k**-point sampling appeared around the turn of the century, which we attribute to this shift of interest.

Recently, the wish to perform highly accurate calculations has raised renewed interest in improved methods for the sampling of reciprocal space. One driving factor originates from computational materials science. For thermodynamic studies, e.g., for the calculation of phase diagrams, highly converged total energies for the elementary unit cells of bulk materials are required (Grabowksi et al., 2007; Grabowski et al., 2011). The necessary calculations should be performed in an automated way, using the methods of high-throughput computing. For this reason, one uses automatically generated, very dense **k**-point sets that allow one to reach an accuracy of the total energy better than 1 meV per atom. As has been shown in a recent study (Morgan et al., 2018), in order to guarantee this accuracy level for *all* phases (with differently sized and shaped unit cells), a **k**-point density as high as 5,000 **k**-points /Å^−3^ is typically required. Moreover, methods based on machine learning attempt to select **k**-point grids that are most suitable for the problem at hand (Choudhary and Tavazza, 2019). As another factor driving innovation in the field of **k**-point sampling, the interest in special properties of bulk materials, in particular in the areas of electronic transport, magnetism and topological states of matter, has lead to improved (e.g., adaptive) schemes. While the total energy is a quantity that is variational with respect to small changes in the charge density, and is thus computationally robust, the applications mentioned above require the resolution of very fine structures in the Brillouin zone in order to obtain an accurate description of the properties of interest.

While we restrict ourselves to density functional theory calculations in this review, an *ab initio* treatment of periodic systems with the wavefunction-based methods of quantum chemistry is an alternative option that is free of any inaccuracies due to approximate density functionals. Recently, this field has attracted strong interest and significant progress has been achieved (Booth et al., 2012; Gruber et al., 2018). Most of the statements about DFT calculations made in this review carry over to the Hartree-Fock approximation, which, like DFT, describes the wavefunction in terms of single-particle orbitals. Efficient computer codes for Hartree-Fock calculations including the option to treat period systems (Dovesi et al., 2018) are available. Post-Hartree-Fock methods incorporate electronic correlations in various approximate ways; examples applicable to periodic systems are the Møller-Plesset perturbation theory or the Coupled-Cluster method (Gruber et al., 2018). Here, the difficulty of treating correlations between two electrons in different unit cells of the solid must be tackled with. The best correlated ground-state wavefunction (in a variational sense) may have fewer symmetries than the many-particle Hamiltonian; therefore approaches exploiting the translational symmetry of the crystal need to be considered with caution. A systematic way to include electronic correlations in calculations for periodic systems is offered by the method of increments (Paulus, 2006). Another option applicable to periodic systems is the Quantum Monte Carlo method (Foulkes et al., 2001) which allows for an even more flexible mathematical representation of the many-particle wavefunction than methods starting from a basis set of atomic orbitals.

This review article serves the purpose of informing researchers in the materials physics community about current developments in the area of high-throughput computing with high demands on the accuracy of atomic structures, forces and total energies. At the same time, we aim at providing a tutorial for newcomers to the field of density functional theory calculations, and therefore give a summary of the basic knowledge about periodic DFT calculations that has been accumulated over the years but is spread out over a bunch of articles in the original literature that are inconvenient to access for the beginner. Since the essentials of the Kohn-Sham approach have been described in a number of textbooks (Parr and Weitao, 1994; Koch and Holthausen, 2001; Martin, 2004; Sholl and Steckel, 2009), we assume that the reader is familiar with them. Convergence issues with respect to the plane-wave expansion of the Kohn-Sham wavefunctions (Kresse and Furthmüller, 1996a) or with respect to atom-centered basis sets (Koch and Holthausen, 2001; Blum et al., 2009) should be addressed by practitioners of DFT before turning their attention to periodic systems; again, we refer to the literature.

## 2. Basics of Crystallography

The periodic arrangement of atoms in a crystal is mathematically described by its smallest periodic unit, the **unit cell**, and by a lattice of points invariant under translations. The lattice points *R* must fulfill the equation

(1) $$\begin{array}{l} \text{R}=n_{1}{\text{a}}_{1}+n_{2}{\text{a}}_{2}+n_{3}{\text{a}}_{3}, \end{array}$$

where *n*_1_, *n*_2_, *n*_3_ are (positive or negative) integers. The **lattice vectors a**_1_, **a**_2_, **a**_3_ span the three-dimensional unit cell with volume Ω.

The unit cell may be occupied by a single or by several atoms; in the latter case, crystallographers call the positions of the atoms within the unit cell the (crystallographic) **basis**. The possible shapes of unit cells are limited by the considerations that the periodic repetitions of the unit cell must be space-filling, i.e., there are no overlaps or voids. The lattices described by Equation (1) that fulfill this condition are called **Bravais lattices**.

For any crystal lattice in general, it must hold that the discrete symmetries (i.e., mirror symmetry or invariance under certain rotations) of the pattern formed by all atoms (including those defined through the crystallographic basis) are compatible with the invariance under translations defined by the Bravais lattice. This excludes e.g., dodecahedra or icosahedra as unit cells. However, it may well be that the basis has a lower symmetry than the Bravais lattice itself. In fact, this leads to a finer classification of crystal structures – the Bravais lattice is just the topmost level in a hierarchical classification scheme (Streitwolf, 1971; Ashcroft and Mermin, 1976).

Crystal symmetry is treated within the mathematical field of **group theory**. The relevant groups (called point groups and space groups) consists of a finite number of symmetry operations *N*_group_. As for any group, the crystallographic groups must be closed under the inclusion of any composite operations, where composite means the sequential application of two discrete symmetry operations (= group elements *G*_α_, α = 1, …*N*_group_) after one another. Under the term **crystallographic point group** one addresses a certain collection of discrete symmetry operations, such as reflections or rotations, that form a group in the mathematical sense and that map (at least) one point of the crystal lattice (which is considered to be infinite for this purpose) onto itself, while any other lattice point may be mapped onto a different lattice point. The concept of the point group does not make reference to translations; if we require the crystal, in addition to invariance under the point group operations, to obey translational symmetry under some symmetry operations *T*_***R***_***n***__, we reach the (more rich) concept of a Bravais lattice. All Bravais lattices having the same set of discrete symmetries, i.e., having the same point group, are said to belong to the same **crystal system**. An example is the cubic crystal system that contains the simple cubic, body-centered cubic (bcc) and face-centered cubic Bravais lattices. The point group, however, may be reduced to a sub-group if the basis is less symmetric than the Bravais lattice itself. The overall number of point groups is therefore higher or equal to the number of Bravais lattices. The actions of the (abstract) symmetry group operations on an electronic wave function ψ(**r**) can be described by linear algebra on real-space vectors, e.g.,

(2) $$\begin{array}{l} {\text{T}}_{{\text{R}}_{n}}\psi \left(\text{r}\right)=\psi \left(\text{r}+{\text{R}}_{n}\right), \end{array}$$

in other words, the position **r** of the electron is shifted by a lattice vector **R**_*n*_. In case of a rotation or reflection, the symmetry operation is represented by some matrix *G*_α_, e.g.,

(3) $$\begin{array}{l} {\text{G}}_{\alpha }\psi \left(\text{r}\right)=\psi \left(G_{\alpha }\text{r}\right). \end{array}$$

If the crystal possesses a certain symmetry, the corresponding symmetry operation acting on the wavefunction changes it by nothing more than a phase factor,

(4) $$\begin{array}{l} {\text{T}}_{{\text{R}}_{\text{n}}}\psi \left(\text{r}\right)=e^{i\phi_{n}}\psi \left(\text{r}\right), \end{array}$$

(5) $$\begin{array}{l} {\text{G}}_{\alpha }\psi \left(\text{r}\right)=e^{i\phi_{\alpha }}\psi \left(\text{r}\right). \end{array}$$

with real numbers ϕ_*n*_ and ϕ_α_.

The symmetry operations mentioned so far, also called **symmorphic** symmetry operations, comprising translations, rotations and reflections, have in common the property that each single of them leaves the crystal (thought to be infinite and unbounded) invariant. One can imagine cases where the crystal is left invariant only by a certain combination of symmorphic symmetry operations. The two cases of these so-called **non-symmorphic** symmetry operations are the glide plane—the crystal remains invariant only under a combined reflection and translation, typically by a fraction of a full lattice vector—and the screw axis – the crystal remains invariant only under a combined rotation and translation, typically by a fraction of a full lattice vector. By the presence or absence of these non-symmorphic symmetries, the classification scheme for crystals can be made even more diverse than with the point groups alone. Hence, the crystal symmetries, including the non-symmorphic ones, must eventually be described by the crystallographic **space groups**.

The paramount importance of symmetry for quantum mechanics is well-known. In application to crystals, this means: The Hamiltonian of the crystal commutes with all elements of the point group. In this context, the group elements are represented by certain operators on a Hilbert space. Consequently the eigenfunctions of the Hamilton operator have specific properties with respect to the application of symmetry operations.

**Bloch's theorem** (Bloch, 1928)

To be specific, let us consider translational symmetry operations. Although the conditions leading to Bloch's theorem can be taken over to many-particle systems by introducing an artificial 'simulation cell' Hamiltonian (see Rajagopal et al., 1995), we restrict our considerations to single-particle eigenfunctions for electrons. From the translational invariance of the crystal, it follows that the electronic wave functions can change only up to a phase factor under translation,

(6) $$\begin{array}{l} \psi \left(\text{r}+{\text{R}}_{n}\right)=e^{i{\text{k}\text{R}}_{n}}\psi \left(\text{r}\right). \end{array}$$

Another (mathematically equivalent) way of stating this requirement is as follows: The wave function is required to consist of a lattice-periodic factor *u*(**r**), which is the same in each unit cell, *u*(**r** + **R**) = *u*(**r**), multiplied by a plane wave,

(7) $$\begin{array}{l} \psi_{\text{k}}\left(\text{r}\right)=e^{i\text{k}\text{r}}u_{\text{k}}\left(\text{r}\right). \end{array}$$

The index **k** is a vector and may be considered a ‘quantum number' characterizing the wave functions of a periodic crystal. **k** is termed crystal momentum. Please note that also *u*_**k**_(**r**) depends on this index, albeit the dependence being weak in most cases.

**Reciprocal lattice**

For every given set **R** of lattice points, we may (at least in a formal mathematical sense) construct a reciprocal lattice spanned by the **reciprocal lattice vectors b**_1_, **b**_2_, **b**_3_. These are defined by

(8) $$\begin{array}{l} {\text{a}}_{i}{\text{b}}_{j}=2\pi \delta_{ij}. \end{array}$$

In practice, the **b**_*i*_ (in three-dimensional space) are obtained as

(9) $$\begin{array}{l} {\text{b}}_{1}=2\pi \frac{{\text{a}}_{2}\times {\text{a}}_{3}}{\det\left({\text{a}}_{1}{\text{a}}_{2}{\text{a}}_{3}\right)}. \end{array}$$

Formulas for the other two reciprocal lattice vectors can be obtained by cyclic permutations of the indices (*i, j, k*) = (1, 2, 3). The denominator contains the volume of the real-space unit cell, Ω = |det(**a**_1_**a**_2_**a**_3_)|. A unit cell of the reciprocal lattice is also called a **Brillouin zone**, see e.g., Ashcroft and Mermin (1976) for its definition. Due to Bloch's theorem (confer Equation 7) it suffices to know the wave functions ψ_**k**_ for crystal momentum **k** within the primitive unit cell of the reciprocal lattice, i.e., within the **first Brillouin zone**^1^. If a translation acts on a wavefunction, its crystal momentum does not change. The actions of a rotation or reflection *G*_α_ on the wavefunction, represented by a real-space matrix multiplication *G*_α_, operates via its inverse in reciprocal space, i.e., it maps $\text{k}\mapsto G_{\alpha }^{-1}\text{k}$.

## 3. Brillouin Zone Sampling

In density functional theory (DFT), the total energy of an atom, molecule or cluster is obtained by summing over contributions from *all* its electrons. Due to the effective single-particle description offered by the Kohn-Sham method in conjunction with Bloch's theorem, the total energy of a crystal can be calculated from the knowledge of the *u*_**k**_(**r**) in Equation (7) by considering the electronic states where **r** varies just *within one* real-space unit cell. This is a tremendous computational simplification. However, Bloch's theorem requires us to calculate wavefunctions for all boundary conditions specified by Equation (6) with **k**-points in the first Brillouin zone. In a finite crystal, the number of terms to be summed over is equal to the number of unit cells in the crystal (cf. Ashcroft and Mermin, 1976). This corresponds to a very fine sampling, and one often speaks of Brillouin zone *integration* since the huge sum and the integral are interchangeable.

So far, nothing would be gained in terms of computational savings. However, since the lattice-periodic part *u*_**k**_(**r**) of the wave function typically only weakly depends on **k**, it is sufficient to sample the Brillouin zone integral at a finite, usually rather small number of points. The numerical techniques to achieve this are based on Fourier quadrature (Froyen, 1989). For concreteness, let us consider some lattice-periodic function *F*(**k**) that may contain implicit dependencies on the eigenvalues ε_*i*_(*k*) and wavefunctions ψ_**k**_(**r**). We assume that this function can be expanded into a finite number of Fourier components, up to some **R**_*m*_ = (*x*_*m*_, *y*_*m*_, *z*_*m*_),

(10) $$\begin{array}{l} F\left(\text{k}\right)=\overset{m}{\underset{j=1}{\sum }}F_{j}e^{i{\text{k}\text{R}}_{j}}. \end{array}$$

The integral of *F*(**k**) over the whole Brillouin zone is given by the lowest Fourier component *F*_0_, and this integral is supposed to be approximated by a finite sum over **k**-points. Since the following considerations concerning the discretization error rely on the vanishing of large Fourier components (beyond **R**_*m*_), they apply in a strict sense to semiconductors and insulators only. For metals, the presence of a Fermi surface where the occupation of the bands changes rapidly from zero to one implies the presence of high Fourier components in *F*(**k**). In order to minimize the discretization error, one introduces so-called *special* **k**-points. In the simplest case of a cubic crystal with lattice constant *a*, one uses equidistant grids of **k**-points that fill the Brillouin zone homogeneously. For instance, let's assume that the grid consists of *N*_*x*_ points in the *x*-direction, then the finite sum in Equation (10) is able to represent the integral exactly, if *N*_*x*_*a* ≥ *x*_*m*_, the first component of **R**_*m*_. In other words, the finite sum is identical to the integral if the function *F*(**k**) was sufficiently smooth and contained only Fourier components that reached out to *N*_*x*_ lattice constants *a* in real space. Even in case of non-cubic unit cells, the **k**-points may still be chosen to lie on planes parallel to the planes spanned by two reciprocal lattice vectors, i.e., parallel to the faces of the unit cell in reciprocal space. If the crystal possesses point symmetries, these symmetry operations can be used to reduce the number of **k**-points for which an actual calculation of the Kohn-Sham wave functions needs to be performed. In other words: It is sufficient to sample only the *irreducible wedge* of the Brillouin zone. This results in considerable savings of computational costs. By virtue of symmetry mapping, one can always 'unroll' the irreducible wedge^2^ to recover the full Brillouin zone. In this way, it is always possible to recover the fully symmetric charge density, forces, etc. when needed. Even if the unit cell has no point group symmetries, time reversal invariance allows to map the wavefunction ψ_**k**_ to $\psi_{-\text{k}}=\psi_{\text{k}}^{*}$, and thus the number of k-points can be reduced by a factor of two. This procedure, using time reversal invariance, is broken by a magnetization or an external magnetic field, and it does not apply if spin-orbit interactions are to be considered in a system that lacks inversion symmetry. If additional point group symmetries are present, the reduction of the number of **k**-points will be even larger, and thus allows for additional savings in computer time. Hence, exploiting symmetries is highly recommendable.

The DFT codes in use also symmetrize the charge density and the forces on the atoms if applicable. This leads to an important *caveat*: Symmetry can *not* be exploited if one wants to investigate symmetry-breaking relaxations of the atoms starting from an “ideal” symmetric structure. In this case, most codes offer an option to switch off the symmetrization manually (with the consequence of significantly enhanced computational resources required). Then, the forces are not symmetrized and the numerical rounding error (or some manually applied small displacement) is sufficient to drive the system from a symmetric starting point to its symmetry-breaking ground state.

### 3.1. Economic Choices of **k**-point Grids

In early computational work, when computer memory was a major limitation, the focus was on an economic choice of **k**-points for Brillouin zone sampling that made it possible to carry out calculations for real-space unit cells with many atoms. In the simplest case, one could estimate the Brillouin zone integral by the value of the integrand at a single point, the so-called Baldereschi point (Baldereschi, 1973). However, for more accurate calculations, a larger set of special **k**-points should be used. The convergence criteria, as well as explicit listings of special **k**-points sets for three-dimensional crystals, have been worked out by Chadi and Cohen (1973). One starts by defining symmetrized functions *A*_*m*_(**k**) that are characteristic of a certain “shell” of **k** vectors. The index *m* can be associated with some length |**k**|, i.e., with some shell of equidistant **k**-points around the Γ-point that marks the origin of the reciprocal lattice. Formally we define

(11) $$\begin{array}{l} A_{m}\left(\text{k}\right)=\overset{N_{\text{group}}}{\underset{\alpha }{\sum }}{\text{G}}_{\alpha }e^{i\text{k}\text{r}}, \end{array}$$

where the summation runs over all elements *G*_α_ in the point group, in other words, it amounts to applying all point group operations onto some plane wave *e*^*i***kr**^ with given **k**. By choosing a set of special **k**-points **k**_*i*_, *i* = 1, …*N*_special_, the aim is to make the leading shells *A*_*m*_ (those for which *F*(**k**) has significant contributions) to vanish, such that we have $\underset{i}{\sum }w_{i}A\left({\text{k}}_{i}\right)=0$ with some suitably chosen weighting factors *w*_*i*_. Any lattice-periodic function *F* can be integrated according to

(12) $$\begin{array}{l} \frac{\Omega }{{\left(2\pi \right)}^{3}}\int_{\text{BZ}}d\text{k }F\left(\text{k}\right)=F_{0}=\overset{N_{\text{special}}}{\underset{i=1}{\sum }}w_{i}F\left({\text{k}}_{i}\right)+\overset{\infty }{\underset{m=M+1}{\sum }}\overset{N_{\text{special}}}{\underset{i=1}{\sum }}w_{i}F_{m}A_{m}\left({\text{k}}_{i}\right). \end{array}$$

Due to the choice of a special **k**-point set, the *M* leading terms in the first sum in the second term vanish exactly. For a smooth function *F*(**k**), the *F*_*m*_ get smaller and smaller as we go to higher and higher shells. Therefore, it is reasonable to drop the second term in Equation (12) as a whole, and estimate the Brillouin zone integral by the first term. The quality of a special **k**-point set can be judged by specifying how many leading shells of *A*_*m*_ it makes to vanish. However, for a final judgement of the accuracy of Brillouin zone integration, one also needs to consider the properties of the function *F* to be integrated. Obviously, some assumptions about the smoothness of *F* are required to reach satisfactory accuracy in the integration, which are fulfilled if *F* stands for the charge density, total energy density, or any other quantity representing the systems as a whole.

**Figure 1:**
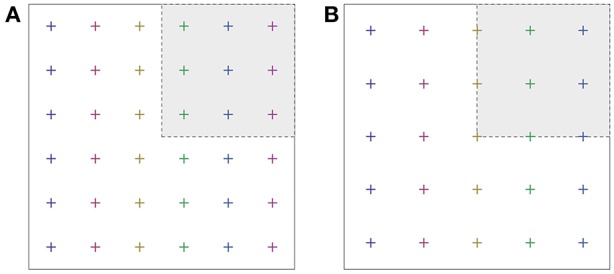
Special **k**-point set according to Monkhorst and Pack (6 × 6, shifted away from Γ) **(A)**, and a Γ-centered grid **(B)**. Note that the fraction of **k**-points falling onto the coordinate axis is larger in the second case.

In terms of computational economy, one important issue is the question whether some special **k**-points come to lie on symmetry planes or symmetry axes (mirror planes, rotational axes), or if they avoid the symmetry-invariant *loci* of the Brillouin zone. For an efficient sampling with some given number of **k**-points, these should be “representative” of any point in the Brillouin zone, and therefore they should *stay away* from the symmetry plane or axes. In this case, all **k**-points would ideally contribute to the Brillouin zone integral with equal weight (Figure 1A). If a special **k**-point falls onto a symmetry plane or axis, it contributes with a smaller weight (Figure 1B). It does not represent so many other **k**-points outside the irreducible wedge, because some symmetry operations map this point onto itself, rather than onto another **k**-point. Grids of special **k**-points with unequal weights *w*_**k**_ tend to be less efficient than a grid with equal weights; however, unequal weights often cannot be avoided completely. Thus, one approximates the Brillouin zone integral of some function *F* by a weighted sum,

(13) $$\begin{array}{l} F_{0}\approx \overset{N_{\text{special}}^{\text{irred}}}{\underset{j=1}{\sum }}w_{j}F\left({\text{k}}_{j}\right). \end{array}$$

The weights (that contain the effects of symmetry) must sum up to unity, $\underset{j}{\sum }w_{j}=1$. The question how efficiently any given set of **k**-points can be reduced due to symmetry has been studied systematically for the lattices of the cubic crystal system by Moreno and Soler (1992). The authors provide tables listing the number of irreducible **k**-points for a given number of **k**-points in the total BZ, and indicate how many shells (the index *M*) in Equation (12) are made to vanish by this choice. More recently, the methods of informatics have been applied to this topic (Wisesa et al., 2016) and a database with **k**-point sets rendering the strongest symmetry reduction for a given lattice have been published. Moreover, a robust algorithm for symmetry reduction of large **k**-point sets has been published recently (Hart et al., 2018).

Nonetheless, sometimes there are reasons why one would like to include a point of high symmetry (e.g., the Γ point) into the special **k**-point set. For instance, the high-symmetry point may be physically important, e.g., if it is the maximum or minimum of the conduction or valence band in a semiconductor. However, even in this case one would prefer to first carry out a self-consistent calculation of the total energy using a **k**-point set without the Γ-point, which then could be followed by a non-self-consistent calculation with a denser **k**-point grid including the Γ point to obtain the density of states or the electronic band structure along high symmetry lines in the Brillouin zone.

In the simplest case, a **k**-point grid is specified by a product of three integer numbers, e.g., a 10 × 10 × 10 grid. The number of *reducible* **k**-points is given by the product of these three numbers (1,000 in the present example). In the actual DFT calculation, only a smaller number of **k**-points, the *irreducible* **k**-points, are used. These are the **k**-points that remain after all equivalences between **k**-points due to point group symmetries and time-reversal symmetry have been exploited. This helps to reduce the required computational resources considerably.

Mathematically speaking, the **k**-point set is expressed by a set of fractional numbers κ, the coordinates of the **k**-points in a coordinate system spanned by the reciprocal lattice vectors **b**_*j*_. For example, for a cubic lattice with lattice constant *a* and a *q*_*x*_ × *q*_*y*_ × *q*_*z*_ grid, one chooses

(14) $$\begin{array}{l} \text{k}=\left(\kappa_{x,r},\kappa_{y,r},\kappa_{z,r}\right)\pi /a\text{ with} \end{array}$$

(15) $$\begin{array}{l} \kappa_{x,r}=\frac{2r_{x}-q_{x}-1}{2q_{x}},\;r_{x}=1,\ldots q_{x}, \end{array}$$

(16) $$\begin{array}{l} \kappa_{y,r}=\frac{2r_{y}-q_{y}-1}{2q_{y}},\;r_{y}=1,\ldots q_{y}, \end{array}$$

(17) $$\begin{array}{l} \kappa_{z,r}=\frac{2r_{z}-q_{z}-1}{2q_{z}},\;r_{z}=1,\ldots q_{z}. \end{array}$$

with integers *r*_*x*_, *r*_*y*_ and *r*_*z*_.

This corresponds to the choice of Monkhorst and Pack (1976) and Pack and Monkhorst (1977). For even *q*_*x*_, *q*_*y*_, *q*_*z*_, the factor 2 in the denominators ensures that the **k**-points stay away from the coordinate axis of reciprocal space. An alternative could be a grid centered around the Γ-point. In this case, one simply chooses κ_*x, r*_ = *r*_*x*_/*q*_*x*_ − 1, … and so on. Here, the **k**-points fall onto the coordinate axes, which, after reduction by the symmetries, leads to unequal weights *w*_*r*_*x*_, *r*_*y*_, *r*_*z*__.

**Figure 2:**
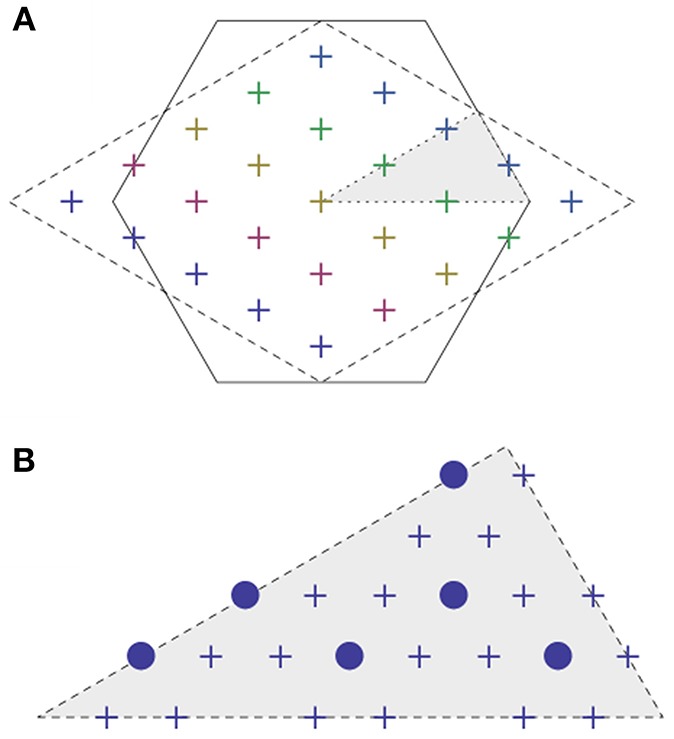
**(A)** First Brillouin zone of the hexagonal lattice with irreducible (shaded) wedge. The conventional, rhombic Brillouin zone used in DFT calculations is indicated by the dashed line, together with a 5 × 5 Monkhorst-Pack mesh. **(B)** Cunningham's special **k**-point set (Cunningham, 1974) consisting of six points (filled circles) or 18 points (crosses) in the irreducible wedge.

Studying two-dimensional **k**-point grids is instructive, because their visualization is easy. Moreover, they are of practical relevance in the study of surfaces (see section 3.5 below). The ideas of Monkhorst and Pack carry over to all five 2D Bravais lattices permitted by surface crystallography. An interesting situation occurs for the hexagonal lattice. The two-dimensional unit cell can be described by a rhombic shape. Following Monkhorst and Pack, the **k**-points are distributed on lines that run parallel to the edges of the rhombic shape. Consequently, a high fraction of the **k**-points falls onto the coordinate axis that bisects the rhombus (Figure 2A). Following the work of Cunningham (1974), there are alternative choices for special **k**-point sets. In his scheme, he starts from a small set of “generating” **k**-points and then arrives at his special **k**-point set by applying the point group operations *G*_α_ to this “generating” set. The procedure can be repeated, thereby obtaining finer and finer **k**-point meshes by starting from increasingly larger generating sets. In this way, he obtains first a set of three, then six, and then 18 *irreducible* **k**-points (see Table 1). When unfolded on the entire Brillouin zone, these yield sets of 18, 54 and 162 **k**-points that cannot by expressed in the usual notation, as *q*_*x*_ × *q*_*y*_, but rather are distributed in a way compatible with the hexagonal symmetry (Figure 2B). Thus, they allow for a high symmetry reduction factor (up to nine).

**Table 1:** Cunningham's choice of special **k**-points in the two-dimensional hexagonal lattice.

***N*_special_**	**${\text{N}}_{\text{special}}^{\text{irred}}$**		
18	3	$\left(\frac{10}{9},\frac{2}{3\sqrt{3}}\right),\left(\frac{8}{9},0\right),\left(\frac{4}{9},0\right)$	
54	6	$\left(\frac{2}{9},\frac{2}{9\sqrt{3}}\right),\left(\frac{4}{9},\frac{4}{9\sqrt{3}}\right),\left(\frac{8}{9},\frac{8}{9\sqrt{3}}\right)$	$\left(\frac{6}{9},\frac{2}{9\sqrt{3}}\right),\left(\frac{8}{9},\frac{4}{9\sqrt{3}}\right),\left(\frac{10}{9},\frac{2}{9\sqrt{3}}\right)$
		$\left(\frac{4}{27},0\right),\left(\frac{8}{27},0\right),\left(\frac{16}{27},0\right)$	$\left(\frac{10}{27},\frac{2}{9\sqrt{3}}\right),\;\left(\frac{14}{27},\frac{2}{9\sqrt{3}}\right),\;\left(\frac{22}{27},\frac{2}{9\sqrt{3}}\right),$
162	18	$\left(\frac{34}{27},\frac{2}{9\sqrt{3}}\right),\left(\frac{32}{27},\frac{4}{9\sqrt{3}}\right),\left(\frac{28}{27},\frac{8}{9\sqrt{3}}\right)$	$\left(\frac{16}{27},\frac{4}{9\sqrt{3}}\right),\left(\frac{20}{27},\frac{4}{9\sqrt{3}}\right),\left(\frac{28}{27},\frac{4}{9\sqrt{3}}\right),$
		$\left(\frac{20}{27},0\right),\left(\frac{18}{27},0\right),\left(\frac{32}{27},0\right),$	$\left(\frac{22}{27},\frac{6}{9\sqrt{3}}\right),\left(\frac{26}{27},\frac{6}{9\sqrt{3}}\right),\left(\frac{26}{27},\frac{2}{9\sqrt{3}}\right)$

*In the 3-point set, all weights w_k_ = 1/3, in the 6-point set the k-points in the third column have w_k_ = 1/9 and the remaining ones in the forth column have w_k_ = 2/9, and in the 18-point set the third column has w_k_ = 1/27 and the forth column w_k_ = 2/27. The location of these k-points is visualized in Figure 2*.

**Figure 3:**
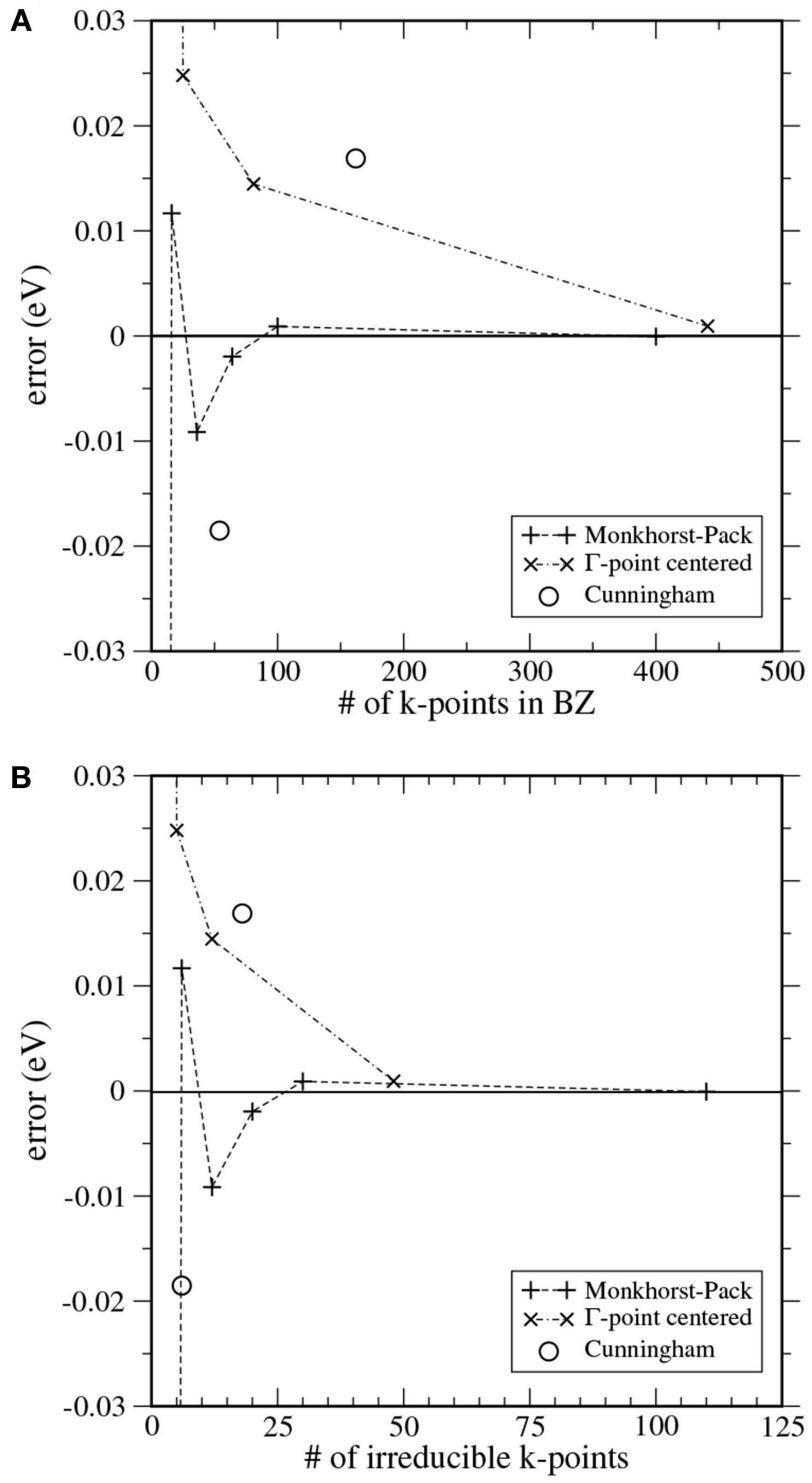
Convergence of the total energy of single-layer graphene calculated with the FHI-aims code (Blum et al., 2009). **(A)** shows the convergence with the total number of **k**-points in the Brillouin zone, corresponding to the inverse **k**-point density, while panel **(B)** shows the scaling with the computational effort, being proportional to the number of irreducible **k**-points, after the operations of the symmetry group *D*_3*v*_ have been applied. The Monkhorst-Pack grids are constructed with *q*_*x*_, *q*_*y*_, *q*_*z*_ in Equation (15)–(17) being even numbers, and consequently the Monkhorst-Pack sets *exclude* the Γ-point.

The convergence of various **k**-point sets for single-layer graphene is compared in Figure 3. The Monkhorst-Pack **k**-point sets (which are defined to avoid high-symmetry points) show fast convergence, both as a function of **k**-point density (Figure 3A) and as a function of the computational effort (Figure 3B). The Γ-centered grids displays a slower, monotonic convergence in the case of graphene, but allow for a stronger symmetry reduction. Note that the hexagonal lattice behaves differently with respect to symmetry reduction from the cubic or orthorhombic lattices discussed above. The calculations with the Cunningham **k**-point sets also benefit from symmetry reduction, but their accuracy is comparable or less than for the Γ-centered and Monkhorst-Pack sets.

For analysis purposes, one often plots the total density of states *g*(ε), defined by

(18) $$\begin{array}{l} g\left(\varepsilon \right)=\underset{i,\text{k}}{\sum }\delta \left(\varepsilon -\varepsilon_{i,\text{k}}\right). \end{array}$$

Obviously, the δ-function in Equation (18) is an example for an integrand *F*(**k**) that is *not* a smooth function of **k**. Another example occurs if the Fermi surface(s) of a metal (see below) should be calculated. In both situations, a very fine sampling of the Brillouin zone is required to obtain accurate results. A way to achieve this is the *tetrahedron method*, originally proposed in its linear form (Jepsen and Andersen, 1971). Within a small tetrahedron, a band ε_*i*_(**k**) can be approximated by a linear function^3^ or by quadratic interpolation of the bands (MacDonald et al., 1979; Blöchl et al., 1994). Moreover, tetrahedra are flexible enough to provide a space-filling coverage (a tessellation) of the whole Brillouin zone. With these pre-requisites, the Brillouin zone integration can be performed over a set of piecewise linear functions. If a band crosses the Fermi energy within one tetrahedron, it will cut out a triangle within this tetrahedron. The entire Fermi surface can be composed of a set of all these triangles. Practical extensions of the method even go beyond the piecewise linear approximation. This is necessary because some electronic bands (those that are partners under some non-trivial representation of the symmetry group operations) display local extrema at the high-symmetry points in the Brillouin zone. Then, it is more adequate to use a quadratic approximation for these bands. Even in the interior of the BZ, bands are usually curved, and one particular (say, positive) curvature may prevail over the Brillouin zone (if e.g., these bands are all derived from the same atomic orbitals). Then, including a quadratic correction may give a systematic improvement of the sampling, as shown e.g., for copper and NiSi_2_ in Blöchl et al. (1994).

### 3.2. Metallic Systems

Density functional theory has initially been proven for atomic and molecular systems with a finite, integer number of electrons. With the help of Bloch's theorem, the proof has been carried over to an infinite, periodic crystal of a semiconducting or insulating material: A finite number of bands, say *N* bands, are fully occupied, separated from unoccupied states by an energy gap. In a spin-polarized calculation, *N* equals the number of electrons in the unit cell; in the (more common) case of a spin-compensated calculation, *N* is only half of the electron count, since each band may host two electrons (of opposite spin). In either case, a numerical calculation can be carried out by considering the occupied bands only, disregarding unoccupied electronic states. This approach, only keeping the occupied bands in the calculations, has been used successfully in many earlier studies, including *ab initio* molecular dynamics, e.g., of liquid water or solid silicon.

For a fully periodic, metallic solid, however, we have to allow for the situation of bands being partially occupied. Hence, the Kohn-Sham formalism needs to be extended to include occupation numbers *f*_*i*_ ∈ [0, 1] (in the spin-polarized calculation; otherwise, *f*_*i*_ ∈ [0, 2] are admissible due to spin degeneracy). Moreover, at finite temperature some electrons in a metal will be in excited states, since there is no energy gap in a metal that could prevent such excitations. From a very general point of view, it has been shown that the validity of DFT can be extended to finite temperatures by the concept of *ensemble DFT* (Mermin, 1965; Marzari et al., 1997), i.e., the electron density now follows from a variational principle derived from a suitably defined *free* energy. This means that the occupation numbers must become functions of temperature, *f*_*i*_(*T*_*e*_) = *f*(ε_*i*_, *T*_*e*_). From a physics point of view, one may prefer to choose the Fermi-Dirac distribution function for the occupation numbers,

(19) $$\begin{array}{l} f\left(\varepsilon ,T_{e}\right)=\frac{1}{\exp\left(\left(\varepsilon -E_{F}\right)/\left(k_{B}T_{e}\right)\right)+1}, \end{array}$$

where *T*_*e*_ is the temperature of the electronic system, *k*_*B*_ is the Boltzmann constant, and *E*_*F*_ is the Fermi energy which can be also regarded as the chemical potential of the electrons μ(*T*_*e*_).

From a numerical perspective, choosing an occupation function is motivated by additional considerations: Since we work with a finite number of **k**-points, the electronic eigenvalue spectrum in a calculation is always discrete. In order to mimic the behavior of a metal, one uses a “smearing” technique, i.e., *T*_*e*_ is set to a high value (typically a few tenth of an eV, i.e., an order of magnitude higher than the physical temperature) to have a smooth transition of the occupation numbers between 1 at low energies and 0 at high energies. This “smearing” allows for numerically stable algorithms to locate the Fermi energy from the set of calculated eigenvalues ε_*i*_(*k*_*j*_). Within the DFT code, the Fermi energy *E*_*F*_ is adapted in each iteration to fulfill the condition

(20) $$\begin{array}{l} \overset{N}{\underset{i=1}{\sum }}\underset{k_{j}\in \text{BZ}}{\sum }f\left(\varepsilon_{i}\left(k_{j}\right),T_{e}\to 0\right)=N, \end{array}$$

where *N* is the number of electrons per unit cell. Likewise, one can define a Fermi temperature by the relation *T*_*F*_ = *E*_*F*_/*k*_*B*_ with *k*_*B*_ the Boltzmann constant. Instead of the summation over the Brillouin zone, one can also use energy integration if one allows for an additional factor *g*(*E*) in the integrand accounting for the density of states,

(21) $$\begin{array}{l} N=\int_{-\infty }^{\infty }dEg\left(E\right)\text{Θ}\left(E_{F}-E\right), \end{array}$$

where Θ is the Heaviside function. After introducing the ‘smearing' parameter σ and performing a transformation to a dimensionless variable

(22) $$\begin{array}{l} x=\frac{E-E_{F}}{\sigma }, \end{array}$$

the Heaviside function Θ(−σ*x*) serves as starting point for various approximations. For instance, it could be replaced by a Fermi-Dirac function with some electronic temperature *T*_*e*_. However, working with an unphysically high *T*_*e*_ introduces an error in the total energy (and also in all other averaged quantities) calculated by the DFT code. In fact, when using *f*_*i*_ = *f*(ε_*i*_(**k**), *T*_*e*_), the code calculates the total energy *E*_tot_(*T*_*e*_) and the free energy *F*_tot_(*T*_*e*_) at finite *T*_*e*_ rather than the total energy *E*_zero_ = *E*_tot_(*T*_*e*_ → 0). The free energy is defined by *F*_tot_(*T*_*e*_) = *E*_tot_(*T*_*e*_) − *T*_*e*_*S*_*e*_(*T*_*e*_) where

(23) $$\begin{array}{l} S_{e}\left(T_{e}\right)=-k_{B}\underset{i}{\sum }\left(f_{i}\text{ln}f_{i}+\left(1-f_{i}\right)\text{ ln}\left(1-f_{i}\right)\right) \end{array}$$

is the electronic entropy. A correction is necessary to obtain the ground state energy at *T*_*e*_ = 0. In the simplest case, one uses the average of the total energy *E*_tot_(*T*_*e*_) and the free energy *F*_tot_(*T*_*e*_), making use of the Sommerfeld expansions (see e.g., Ashcroft and Mermin, 1976, Appendix C)

(24) $$\begin{array}{l} F_{\text{tot}}\left(T_{e}\right)=E_{\text{tot}}\left(0\right)\left[1+a_{2}\frac{T_{e}^{2}}{T_{F}^{2}}+\text{O}\left({\left(\frac{T_{e}}{T_{F}}\right)}^{4}\right)+\ldots \right], \end{array}$$

(25) $$\begin{array}{l} E_{\text{tot}}\left(T_{e}\right)=E_{\text{tot}}\left(0\right)\left[1-a_{2}\frac{T_{e}^{2}}{T_{F}^{2}}+\text{O}\left({\left(\frac{T_{e}}{T_{F}}\right)}^{4}\right)+\ldots \right]. \end{array}$$

**Figure 4:**
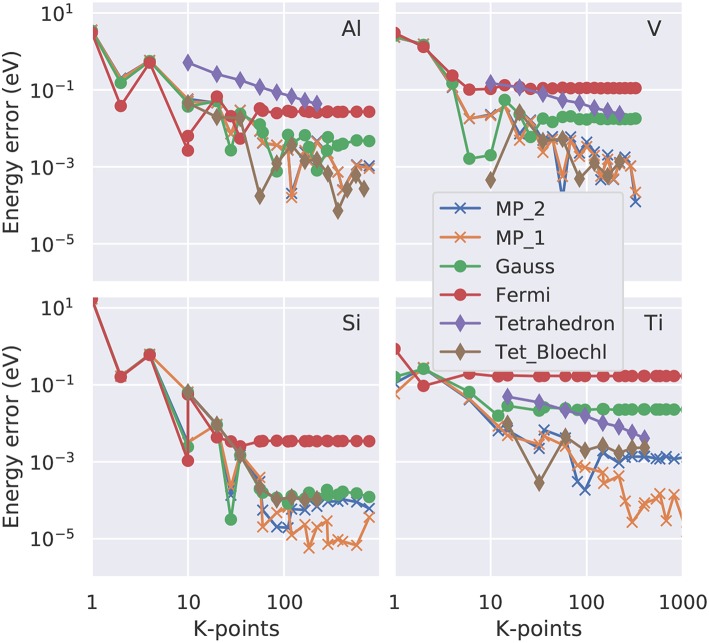
Convergence of the free energy (total energy minus *T*_*e*_*S*_*e*_ with *S*_*e*_ the electronic entropy defined in Equation 23) as function of the number of irreducible **k**-points in a double logarithmic representation. Four representative elements are shown: fcc Al as example for a free electron gas, bcc V with a partially occupied d band, Si as semiconductor and Ti with a hcp crystal structure. The colored lines (see legend) represent the various smearing approaches. “Tet_Bloechl” is the tetrahedron method with Blöchl correction.

**Figure 5:**
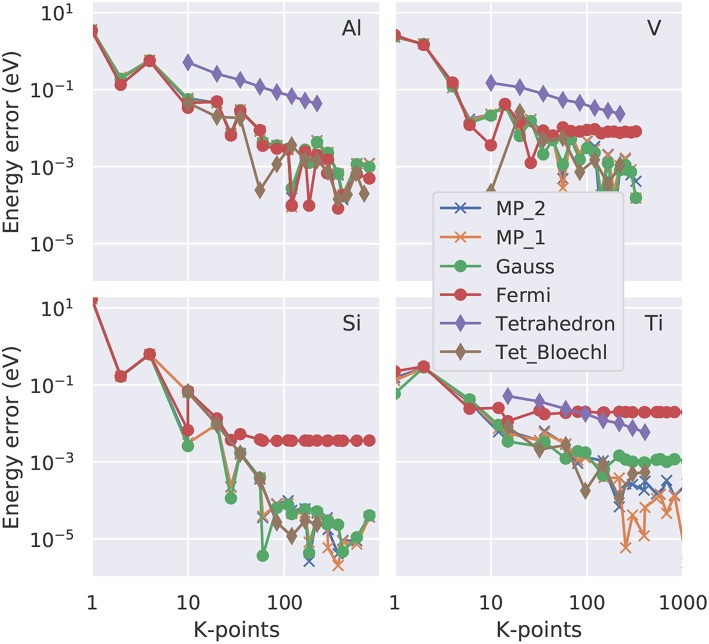
Convergence of the zero energy (total energy extrapolated to *T*_*e*_ = 0K; Equations 26 or 28) as function of the number of irreducible **k-**points in a double logarithmic representation. Four representative elements are shown: fcc Al as example for a free electron gas, bcc V with a partially occupied d band, Si as semiconductor and Ti with a hcp crystal structure. The colored lines (see legend) represent the various smearing approaches. 'Tet_Bloechl' is the tetrahedron method with Blöchl correction.

**Figure 6:**
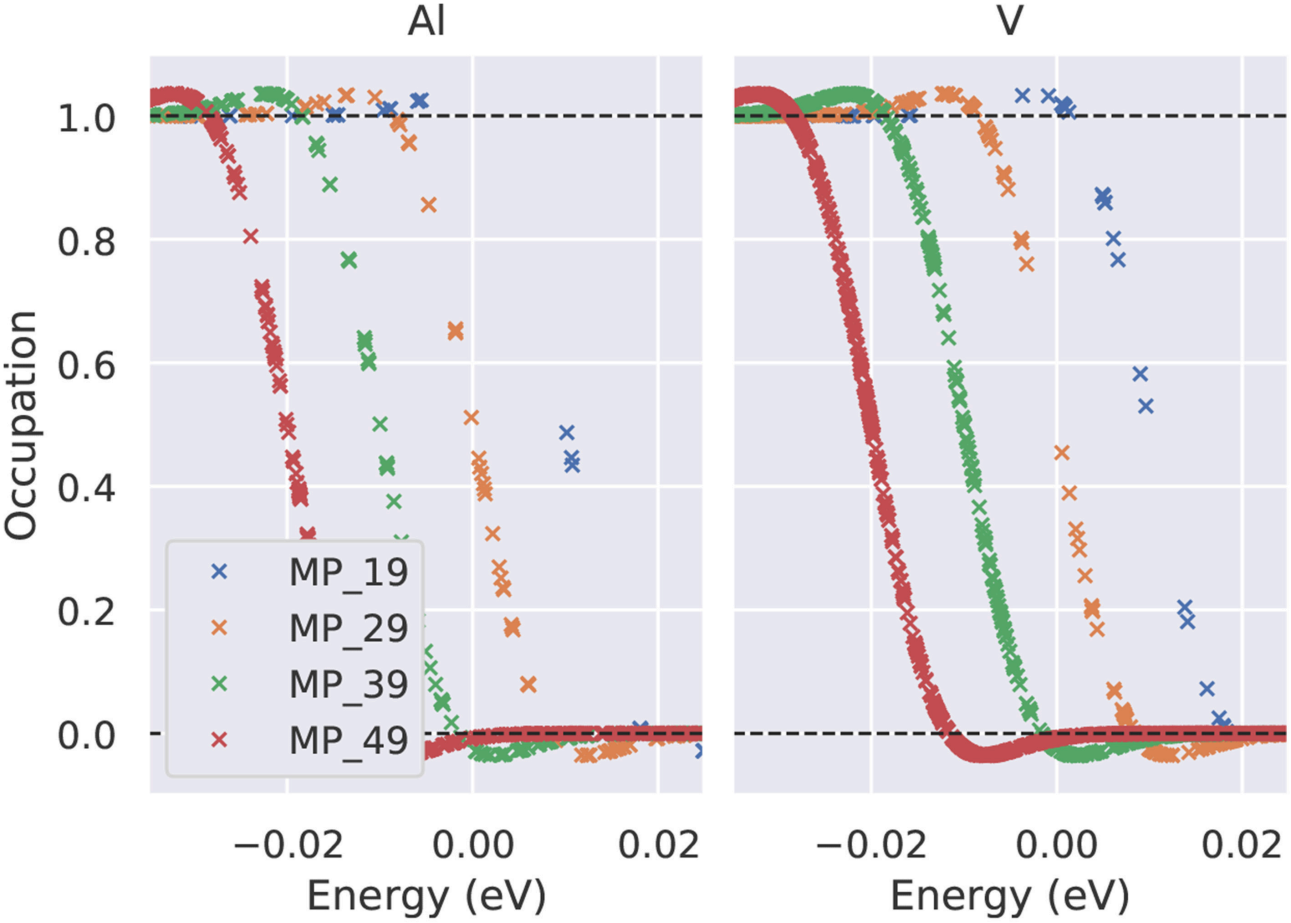
Occupation number of the electronic states close to the Fermi level using the Methfessel-Paxton first-order method and a smearing parameter σ = 0.01eV. The numbers in the legend give the Γ-centered MP mesh (19 indicates e.g., a 19 × 19 × 19 Monkhorst-Pack mesh). For clarity, the curves have been shifted by 0.01 eV each.

The absolute value of the coefficient *a*_2_ in both expansion must be equal, since basic thermodynamics tells us that *F*_tot_ = *E*_tot_ − *T*_*e*_*S*_*e*_, but also *S*_*e*_ = −∂*F*_tot_/∂*T*_*e*_. For instance, in the free electron gas, $a_{2}=\frac{5\pi^{2}}{12}$. If the higher-order terms in the expansion are negligible, since the leading $T_{e}^{2}$ terms cancel each other,

(26) $$\begin{array}{l} E_{\text{zero}}\approx \frac{E_{\text{tot}}\left(T_{e}\right)+F_{\text{tot}}\left(T_{e}\right)}{2} \end{array}$$

yields an improved estimate for *E*_zero_. The behavior of *F*_tot_(*T*_*e*_) and *E*_zero_ will be illustrated by examples in Figures 4, 5. The above equation also gives us a hint how to choose *T*_*e*_ in an actual calculation: It must not be too large (such that the $T_{e}^{4}$ terms are indeed negligible), but at the same time it should be large enough to “smear out” any (unphysical) gaps in the eigenvalue spectrum ε_*i*_(*k*_*j*_) (see e.g., Figure 6). If the latter condition is not satisfied, one must repeat the calculation with a denser grid of **k**-points. Thus, for a metal, the density of the **k**-point grid and the choice of the “smearing" parameter should always be discussed together if highly converged results are needed.

**Figure 7:**
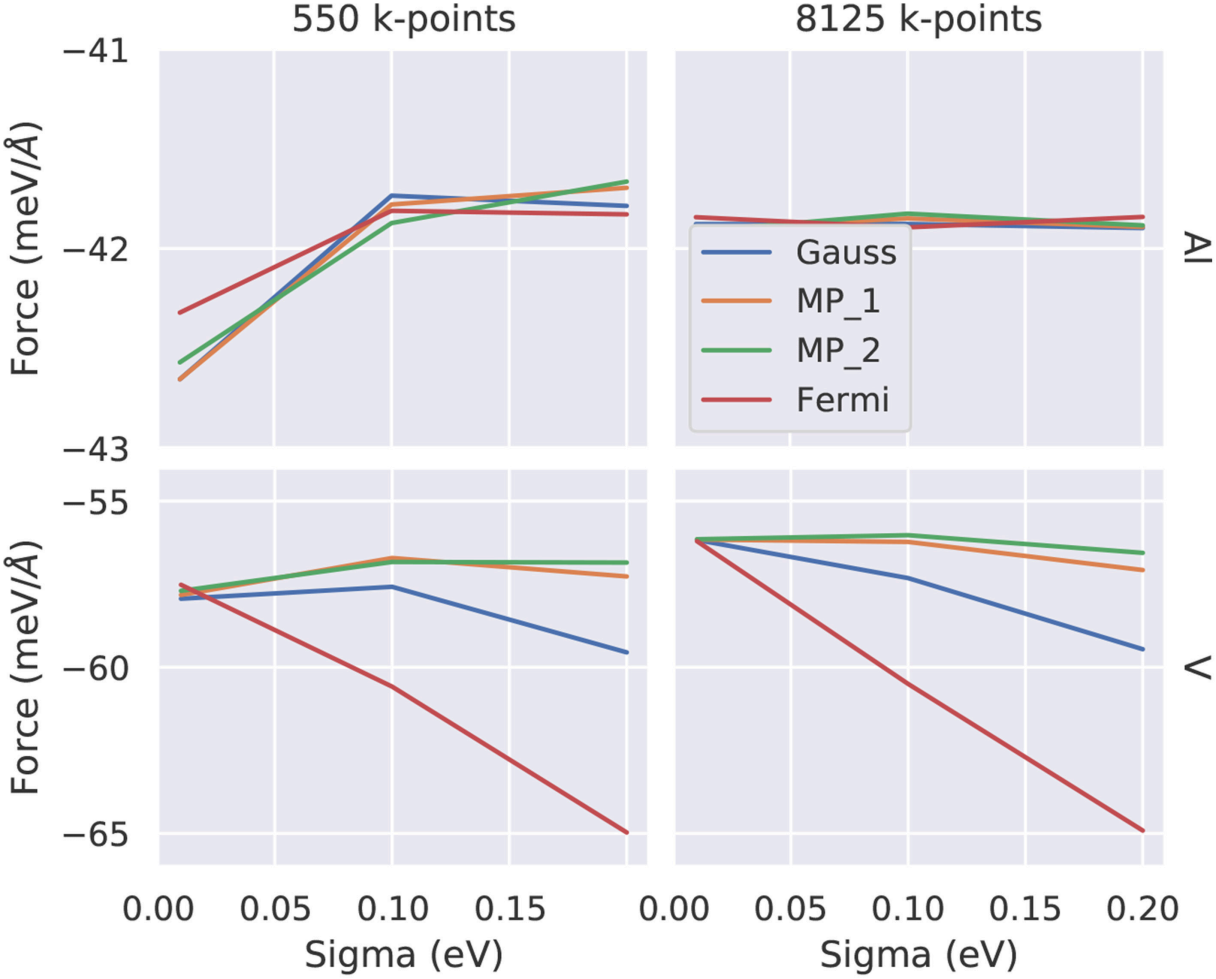
Convergence of the atomic force as function of the smearing parameter σ for two representative elements (top: Al, bottom: V) and for two different **k-**point samplings (left: 550, right: 8125 irreducible **k**-points). The colors indicate the various smearing methods discussed in the text (Gauss, Fermi-Dirac, Methfessel-Paxton first/second order MP_1/MP_2). The converged values are −41.9 meV/Å for Al and −56 meV/Å for V. Note the different *y*-scales indicating a faster **k**-convergence of Al compared to V.

One has to keep in mind that the correction scheme (Equation 26) is applied to the total energy only, while other physical quantities, e.g., the forces on the atoms, are derivatives of the free energy and remain uncorrected. The demand for accurate forces (consistent with the conservation of total energy) is of particular importance in molecular dynamics simulations on the Born-Oppenheimer potential-energy surface and has inspired extensions of the above correction scheme (Wagner et al., 1998) to the calculation of forces. To keep things simple, however, one would like to have a numerical scheme in which already the coefficient *a*_2_ of the leading $T_{e}^{2}$ term is as small as possible. One way to achieve this is the scheme suggested by Methfessel and Paxton (1989). They replace the Fermi-Dirac distribution function (Equation 19) by a complementary error function (erfc) plus an expansion into Hermite polynomials *H*_*n*_(*x*):

(27) $$\begin{array}{l} S_{L}\left(x\right)=\frac{1}{2}\text{erfc}\left(x\right)+\overset{L}{\underset{n=1}{\sum }}B_{n}H_{2n-1}\left(x\right)\exp\left(-x^{2}\right), \end{array}$$

with the variable *x* defined in Equation (22). The function *S*_*L*_(*x*) can also be understood as an approximation to the Heaviside function Θ in Equation (21). The coefficients *B*_*n*_ must be chosen such as to minimize the truncation error of the expansion. In practice, it is advisable to keep only the first few terms in the expansion, *L* = 1 or 2. Choosing *L* = 0 yields the limit of Gaussian smearing, in which the derivative of the Fermi-Dirac function is approximated by a Gaussian function centered at the Fermi energy. It turns out that, with the Methfessel-Paxton scheme with a carefully chosen width σ, not only the total energy, but the forces are sufficiently accurate. This will be discussed later (Figure 7). It is still possible (but not necessary) to extrapolate the total energy to zero broadening using the formula (Kresse and Furthmüller, 1996a)

(28) $$\begin{array}{l} E_{\text{zero}}\approx \frac{E_{\text{tot}}\left(T_{e}\right)+\left(L+1\right)F_{\text{tot}}\left(T_{e}\right)}{L+2} \end{array}$$

which can be seen as a generalization of Equation (26) to the case *L* > 0. The price to be paid when using the Methfessel-Paxton scheme is the possible occurrence of negative *f*(ε_*i*_(*k*_*j*_)), i.e., the ‘occupation numbers' fall outside their physically meaningful interval [0, 1]. An alternative technique, the so-called cold-smearing of the occupation numbers (Marzari et al., 1999), avoids the issue of negative occupation numbers, while it allows for a subtraction technique to remove the entropy contributions from the free energy in order to obtain a corrected expression for the total energy.

**Figure 8:**
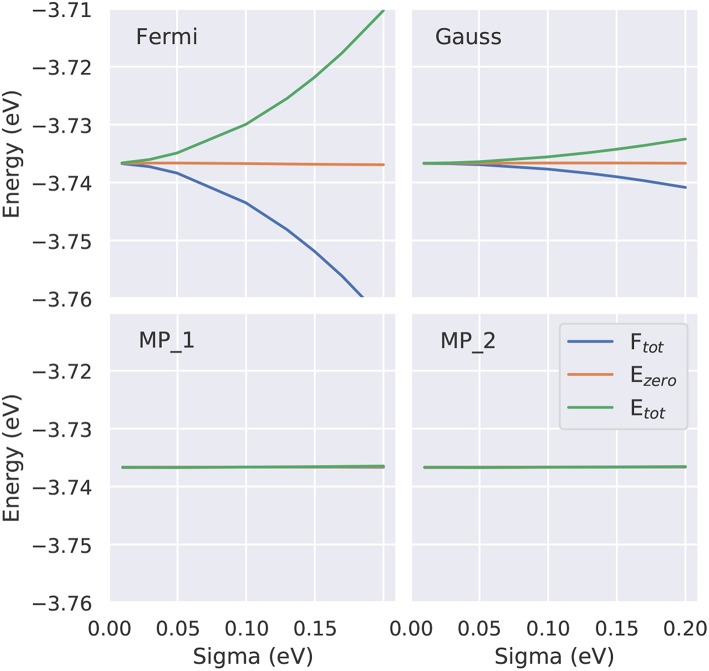
Dependence of the three energies - total energy *E*_tot_, zero energy *E*_zero_ and free energy *F*_tot_ - as function of the smearing parameter for four approaches (Fermi-Dirac, Gauss, Methfessel-Paxton (MP) first and second order). To demonstrate the performance of the MP approaches the identical *y* scaling is used. On this scale for the MP approaches all three energies are identical, showing that even at larger electronic temperatures *T*_*e*_ (i.e., larger σ values) *F*_tot_(*T*_*e*_ = 0K) can be accurately approximated by *E*_zero_(*T*_*e*_).

Test calculations demonstrating the efficiency of the Methfessel-Paxton scheme are shown in Figure 8. While the extrapolation *T*_*e*_ → 0, Equation (26), is necessary to render the Fermi-Dirac or Gaussian broadening independent of the broadening parameter σ, the Methfessel-Paxton scheme gives practically identical values for all three quantities even for σ values as large as 0.2 eV. Further tests for the bulk metals fcc Ru and bcc Ir can be found in Zhang et al. (2017), and for alkali atom adsorption on the Al(111) surface in Neugebauer and Scheffler (1992).

One should keep in mind that there are some topics in solid-state physics that are more sensitive to the sampling of the Brillouin zone than others. Critical issues may arise in particular in cases where a symmetry-lowering phase transition in a material is driven by changes in the electronic structure. For instance, in materials that display an instability against the formation of charge density waves due to Fermi surface nesting, the **k**-point set should be chosen carefully to include the nesting vector. Further examples are the Jahn-Teller effect in polar materials or a Peierls transition, that both may be related to bands that are occupied and/or unoccupied only in small parts of the Brillouin zone; nonetheless it is important to resolve these 'puddles' of electrons and holes within the **k**-point sampling since their extension is driving the structural transition one wants to study.

### 3.3. High-Precision Calculations for Metals

If the total-energy calculations are intended to be used as the basis of a thermodynamics analysis, highly precise values, with an accuracy of 1 meV per atom or better (Grabowksi et al., 2007; Grabowski et al., 2011), are required. Test calculations performed in an automated way for **k**-point grids of various shapes and densities (Morgan et al., 2018) suggest that an accurate sampling of the Fermi surface, and thus a dense **k**-point grid in the range of 30^3^ irreducible **k-**points per atom, is necessary to achieve this goal. Here, we present case studies carried out with the VASP code (Kresse and Furthmüller, 1996b) for four representative materials, aluminum (Al), vanadium (V), silicon (Si), and titanium (Ti). For the dense **k**-point meshes used in this study (e.g., 30 × 30 × 30), it turns out the **k**-point *density* is the decisive quantity, while the exact placement of the grid (e.g., if it contains the Γ-point or not) is a minor issue once a sufficiently high density is used. We plot the absolute error (relative to the **k**-point mesh with the highest density used) as a function of the number of irreducible **k**-points in a double-logarithmic representation both for the free energy (Figure 4) and for the total energy extrapolated to zero broadening (Figure 5). For all materials, using a Fermi-Dirac function, Equation (19), for the occupation numbers does *not* allow for a reliable extrapolation of the total energy to zero temperature, or, more precisely, it converges to a different limit (above the 1 meV threshold) even for the highest **k**-point density used. For metals, the Gaussian smearing of the Fermi edge introduces a too high free energy on the dense grids (see Figure 4); and this error is only insufficiently corrected by the extrapolation to zero broadening (Figure 5). The same statement can be made for the uncorrected (linear) tetrahedron method (Jepsen and Andersen, 1971). The Methfessel-Paxton scheme, both in its first and second order variant, allows to bring down the error to a few meV per atom. The corrected tetrahedron method (Blöchl et al., 1994) performs similar to the Methfessel-Paxton technique, with slight advantages in the range of medium sized **k**-point sets (< 100 irreducible **k**-points), but yields no decisive improvement if a dense **k**-point set is used.

For structural optimization in unit cells that contain more than one atom per cell, the accuracy of the forces is important, too. As a general advice, it is recommendable to use a narrow broadening width if possible, such that the forces are not “contaminated” by unphysical smearing of the electronic eigenvalues and the work done by the forces in a molecular dynamics simulation remains consistent with changes of the total energy. For this reason, it is also advisable to work with the Methfessel-Paxton scheme with first or second-order corrections (*L* = 1 or 2) since its free energy (of which the derivatives are taken to obtain forces) is close to the total energy, cf. Figure 8, lower row. An analysis of the accuracy of forces as a function of the broadening parameter σ is presented in Figure 7 for Al and V (rows) and for a sparse and a dense **k**-point set (columns). Comparing the results of the two **k**-point sets, one finds that using the Methfessel-Paxton scheme with a relatively large broadening, e.g., 0.2 eV, gives reliable results^4^. For a dense **k**-point set, the Methfessel-Paxton results become independent of the value of σ over a wide range. The Gaussian or Fermi-Dirac function broadening are not recommendable since the forces are sensitive to the broadening used. When working with small σ, one should first make sure that the **k**-point set used indeed provides a sufficiently dense sampling of the density of states near the Fermi level. Only then the (numerical) Fermi energy is sufficiently well defined – this can be concluded from the occupation numbers dropping from one to zero gradually, with several fractional occupation numbers on the way, as observed for the green and red curves in Figure 6. In some materials with steep bands crossing the Fermi energy, e.g., in aluminum, this issue deserves special attention. As can be seen from Figure 7, a grid of 19 × 19 × 19 **k**-points is not yet sufficient to define the Fermi energy, because only few occupations *f*_*i*_ ~ 0.5 exist. With these settings (and a small σ = 0.01 eV), also the forces show noticeable deviation from their converged values (see Figure 7, upper left panel). The remedy in this case is provided by using a denser set of 8125 irreducible **k**-points (upper right panel of Figure 7) rather than only 550 irreducible **k**-points (upper left panel of Figure 7).

We note here that the study of realistic materials at finite temperature requires to take *all* physical contributions to the free energy into account. This not only comprises the contribution of electronic excitations to the free energy, but also contributions from lattice vibrations or from magnetic excitations (in case the material displays magnetic order). The “1 meV per atom” accuracy goal not only refers to the electronic free energy, but must also be met by the free energy of the excitations. For the lattice contribution, this requires accurate calculations of phonon spectra and an evaluation of their free energy including anharmonic effects (Grabowksi et al., 2007). Some more technical details how to achieve this goal using a plane-wave DFT code will be given in the next section.

### 3.4. Specialities for Plane-Wave Basis Sets

In plane-wave codes using large basis sets, it can be efficient to evaluate not only the electronic density, but also the expansion coefficients of the wave functions via an iterative scheme. Due to the huge number of basis functions, a full diagonalization of the Hamiltonian for a given (preliminary) electronic density may not be advisable. Rather, one works with approximate, iteratively improved electronic eigenvalues. In this situation, also the occupation numbers *f*(ε_*i*_(**k**_*j*_)) will be approximate as long as the iteration cycle is not yet converged. A method for simultaneous iteration of *both* the wavefunction expansion coefficients *and* the occupation numbers is described in Gillan (1989) and Freysoldt et al. (2009).

Another practical aspect concerns the interplay between atomic relaxation and **k**-point sampling. While relaxation of atomic positions (using the calculated Hellmann-Feynman forces) is still going on, the band occupancies may still change. Smooth changes are preferred for technical reasons – one would like the atoms to move on a smooth (albeit approximate) potential energy surface to be able to use sophisticated algorithms to locate minima and saddle points. To this end, a 'smearing' technique, e.g., Methfessel-Paxton, is appropriate, whereas an accurate representation of the Fermi surface (by the tetrahedron method) is not helpful as it would lead to abrupt changes in the forces if a band (during the course of atomic relaxation) passes through the Fermi energy (becomes populated or depopulated in the next atomic configuration). Moreover, the corrected tetrahedron method (Blöchl et al., 1994) can be shown (Kresse and Furthmüller, 1996a) to result in inconsistencies between the calculated forces and the total energy surface on which the atom or ions are moving. For these reasons, the tetrahedron method is used in the plane-wave community mostly for post-processing of an already converged self-consistent calculation.

When using plane-wave codes, one is typically working with *relative* convergence of energy *differences* with the number of plane-wave basis functions, e.g., convergence of the cohesive energy of a solid (being the difference of the total energies of the crystal and the individual isolated atoms). Absolute convergence of total energies would require an unnecessarily large plane-wave basis set. Under these conditions, special caution is required if one attempts to compare the total energies of two unit cells with different size, or if one wants to calculate the stress acting on a (deformed) unit cell. The number of plane waves in the basis set may change abruptly even under slight changes of the unit cell. As long as absolute convergence has not been reached, this implies a change of the basis set quality that leads to an artificial (unwanted) change in the total energy. One way to correct for such errors is obtained from the scaling hypothesis for finite-basis set corrections (Rignanese et al., 1995). Once the sensitivity of the total energy to the applied plane-wave cut-off has been determined, a correction can be applied that depends both on the cell volume and on the number of **k**-points used to sample the Brillouin zone. The corrected total energy allows one to obtain smooth curves for the volume dependence or strain dependence of physical observables, such as the pressure or the elements of the stress tensor.

Many modern plane-wave calculations use ultrasoft pseudopotentials or the projector-augmented wave (Blöchl, 1996; Kresse and Joubert, 1999) (PAW) method. While these methods are very powerful and robust for most applications, special care must be taken if one attempts to compare the total energies of two atomic configurations that differ only by a slight atomic displacement. This situation is encountered when phonon spectra are calculated via the frozen phonon method. In both the ultrasoft-pseudopotential and the PAW methods, auxiliary charges associated with each atom are stored on a dense real-space grid (within the DFT code). Displacements of the atoms with respect to this grid lead to slight changes in the representation of the augmentation charge that show up as small errors of the total energy that normally go unnoticed. However, when phonon properties are calculated, it is recommended to check the results for convergence with respect to the auxiliary grid density (Grabowksi et al., 2007). The Grüneisen parameter, which is indicative of anharmonic effects, is particularly sensitive to convergence issues. In addition, the accuracy of **k**-point sampling needs to be monitored as well in order to ensure that the tiny energy differences that form the basis of the calculations of phonon frequencies are converged. Such convergence issues are particularly relevant for soft metals with low-frequency phonon modes (e.g., Pb, In) or for the acoustic phonon branches near the Γ-point. Inaccuracies may show up as unphysical modes with imaginary frequencies.

### 3.5. Supercell Model for Surfaces

The modeling of surfaces of a periodic crystal poses various challenges for first-principles calculations. First of all, it is advantageous to retain the two-dimensional periodicity within the surface plane^5^. In most DFT calculations one prefers to use a slab, i.e., a finite number of parallel atomic layers, to model a surface. One may use both surfaces of the slab, front and back side, to calculate the property of interest, e.g., the surface energy, if both sides are equivalent. Otherwise, one uses only one side (the front side) to model the physics of the surface, while the back side is saturated by some atoms, e.g., natural hydrogen or artificial pseudo-hydrogen atoms with fractional nuclear charge, cf. Kaminski et al. (2017). This is commonly done for semiconductor or insulator slabs, while for metal slabs passivation is not needed. For semiconductors the passivation is needed to remove partially occupied electronic backside surface states from the band gap which without passivation could cause a spurious charge transfer through the slab to the surface of interest. For metals, the efficient electronic screening prevents such a transfer, making passivation unnecessary.

In principle, there is no periodicity in the direction of the surface normal. Some DFT codes enable the user to model restricted periodicity to just two (surface) or one (nanowire) dimensions. In codes that use atom-centered localized basis sets, it is natural to discard any wave function overlap in the third dimension of the surface normal. This way of treating just a single slab also helps to save computer time. In plane-wave codes, in contrast, basis functions that are periodic on all three spatial dimensions are used. This means that one is working with a supercell containing a piece of the slab that is periodically repeated. In order to eliminate unwanted interactions between periodic copies of the repeated slab, one needs to include a sufficiently large vacuum region. The thickness of this vacuum region is a parameter that should be tested in each case. As a starting point, one typically uses 10–15 Å of vacuum and checks for the charge density to become zero within the vacuum region. It is important to note that the dispersion of the energy bands, ε(*k*_*x*_, *k*_*y*_, *k*_*z*_), in a slab calculation must vanish in the direction normal to the surface (typically this is the *k*_*z*_ direction). It is clear that the electrons cannot propagate through the vacuum layer, and hence there is no *k*_*z*_ dependence. Therefore, one uses a two-dimensional **k**-point grid in the (*k*_*x*_, *k*_*y*_) plane and sets *k*_*z*_ = 0.

If inequivalent front and back sides of the slab are used, the workfunctions of these two surfaces are generally different, and therefore the electrostatic potential in the vacuum must reach different values depending whether we sample it at a position far outside the front or the back surface. Several DFT codes allow the user to account for this physically meaningful possibility by including a jump of the electrostatic potential with adjustable magnitude right in the middle of the vacuum region. This jump corresponds to the electrostatic potential of an infinitesimally thin dipole layer positioned in the middle of the vacuum region, which is exactly balanced by a dipole density of equal size, but opposite sign, built up by the electronic density inside the slab. This feature, which allows the user to obtain more accurate results for inequivalent surfaces, is called the dipole correction (Neugebauer and Scheffler, 1992).

In the supercell method in general^6^, the cell dimensions in real space are integer multiples *n*_*x*_, *n*_*y*_, *n*_*z*_ of the lattice vectors of the primitive unit cell. Therefore, the reciprocal lattice vectors of the supercell are fractions of the primitive **b**_*i*_ defined in Equation (9). The Brillouin zone of the supercell is smaller by factors *n*_*x*_, *n*_*y*_ and *n*_*z*_ in the respective directions. For this reason, the **k**-point grid in the supercell calculation can be chosen much coarser while retaining the same level of precision. For instance, if the bulk calculation used a *q*_*x*_ × *q*_*y*_ × *q*_*z*_ grid, as a rule, the surface slab calculation with *n*_*x*_ × *n*_*y*_ supercell would use an (equivalent) *q*_*x*_/*n*_*x*_ × *q*_*y*_/*n*_*y*_ grid. The decisive quantity is the *density* of the **k**-points in the BZ, in other word, the volume element or areal element represented by one **k**-point. This technique can be used when differences between surface and bulk total energies need to be calculated, e.g., for the calculation of surface energies.

However, there are exceptions from this rule where the surface requires a denser **k**-point sampling than the bulk. We mentioned already the different demands for sampling density in semiconducting vs. metallic systems. Even if a material is semiconducting in the bulk, it may possess surface or interface states that are metallic. In this case, the “Fermi lines” in the 2D Brillouin zone need to be determined, a task that generally requires a denser sampling of the surface Brillouin zone. One example where the convergence with the **k**-point grid has been documented is provided by the Si(100) surface (Ramstad et al., 1995): On this surface, Si atoms form dimers which have a tendency to tilt with respect to the surface plane. Surface reconstructions with different unit cells parallel to the surface [*p*(2 × 2) or *c*(2 × 2) ] can be formed if the tilting angles on neighboring Si dimers alternate in sign. The energy difference of these reconstructions is very small, demanding a careful sampling technique to obtain accurate results. Moreover, all these surface reconstructions display electronic surface states in the principal band gap of Si. Depending on the relaxed positions of the surface atoms, these surface states may cross the Fermi level, giving rise to parts of the Brillouin zone where an additional electronic state is occupied, and other parts where some other electronic state is unoccupied. These “puddles” of electrons and holes are bounded by Fermi lines. Obviously, we must require some fraction of our **k**-point set to fall into these small parts of the Brillouin zone, only then we will be able to sample the total energy correctly, which is also a prerequisite to obtain the correct relaxed ground state geometry. It has been found (Ramstad et al., 1995) that at least 8 **k**-points in the total BZ of the (2 × 2) cell are required to stabilize the tilting of the Si dimers. Only by using at least 32 **k**-points one can assure that the energy difference of just 5 meV per dimer between the *p*(2 × 2) and the *c*(2 × 2) surface is converged. The importance of partial occupation of bands in a small part of the BZ for driving surface reconstructions is seen even more clearly in the case of gold-induced reconstructions on the Si(111) surface (Erwin et al., 2009). In particular, the dependence of the surface energy on gold coverage, related to the degree of filling of the partially occupied bands, requires careful sampling of the BZ. Inhomogeneous **k**-point grids, with finer sampling in the physically most important areas of the BZ, may be required to accurately describe the observed symmetry-breaking reconstructions.

The number of layers in the slab that is used to model the (in principle) semi-infinite piece of the bulk crystal is another modeling parameter that needs to be chosen carefully. Unfortunately there is no simple rule-of-thumb. For the physical quantity one is interested in, the convergence with slab thickness needs to be checked on a case-by-case basis. For metals, the convergence with thickness is usually faster than for semiconductor or insulator slabs due to the good screening properties of the metal. However, some special surface features, e.g., electronic surface states, may display a very slow decay into the bulk of the crystal. If the physical effects of surface states matter for a particular problem, it may be required to use rather thick slabs of 30 layers or more. One should also keep in mind that a slow convergence or even oscillations of the surface energy as function of slab thickness can be due to physical reasons: For some thin metals films, e.g., Al films (Kiejna et al., 1999) or Pb films (Wei and Chou, 2002), quantum well states occur that induce periodic changes not only of the surface energy, but also of the Fermi level position, work function and other physical quantities.

## 4. Analysis Tools

**Figure 9:**
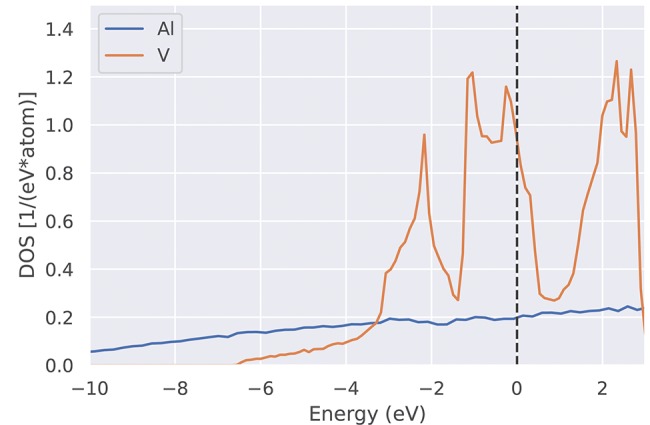
Electronic density of states *g*(ϵ) (DOS) for two representative elements (Al and V). While Al shows a smooth and rather low density of states, the partially occupied 3d bands in V lead to a peaked density of states. The much higher density of states of V at the Fermi level (vertical dashed line) compared to Al explains the denser sampling shown in Figure 6 (see e.g., blue crosses).

The total density of states *g*(ε) introduced in Equation (18) provides information at a glance if a given system is a semiconductor, a metal, a semi-metal or a ferromagnetic half-metal. Therefore, many DFT codes come with a software tool to plot the DOS. In practice, the δ-function in Equation (18) is often replaced by a Gaussian, whose width must be chosen by the user via a separate input parameter to the DFT code. This feature should not be confused with the Gaussian broadening σ discussed in section 3.2. For narrow-gap semiconductors, care must be taken not to close the gap by choosing an unsuitably large Gaussian width. As an alternative, one can plot a histogram of the energy eigenvalue distribution, sorting the eigenvalues into sufficiently narrow bins, as was done in Figure 9, to achieve a graphical representation with sharp features.

The spatially resolved *local* density of states (LDOS) is given by

(29) $$\begin{array}{l} \rho \left(\text{r},\varepsilon \right)=\underset{i,\text{k}}{\sum }|\psi_{i,\text{k}}\left(\text{r}\right){|}^{2}\delta \left(\varepsilon -\varepsilon_{i,\text{k}}\right). \end{array}$$

As one application, the band gap in a semiconductor and the range of surface states can be estimated by producing an LDOS plot of ρ(*z*, ε) along the surface normal of a slab calculation. In this way, one can visualize how the surface states decay into the bulk. The simulation of images of the scanning tunneling microscope (STM), following the Tersoff-Hamann approximation (Tersoff and Hamann, 1983), is another application of the LDOS. Here, one generates a 2D plot in the (*x, y*)-plane (parallel to the surface) of the quantity $\int_{E_{F}}^{E_{F}+V}\rho \left(x,y,z_{\text{tip}},\varepsilon \right)d\varepsilon$ where *z*_tip_ is the position of the STM tip above the surface and *V* is proportional to the voltage applied between tip and sample.

The *atom-projected* density of states (PDOS) can be used to assign the density of states to specific layers (at or below the surface), or specific atomic orbitals:

(30) $$\begin{array}{l} \rho_{M,l}\left(\varepsilon \right)=\underset{i,\text{k}}{\sum }|\int \Phi_{M,l}^{\text{at}}\left(\text{r}\right)\varphi_{i,\text{k}}\left(\text{r}\right){|}^{2}\delta \left(\varepsilon -\varepsilon_{i,\text{k}}\right)d\text{r} \end{array}$$

Here $\Phi_{M,l}^{\text{at}}\left(\text{r}\right)$ stands for an atomic orbital with angular momentum *l* at the atom labeled *M*. The radial dependence of this orbital and its possible truncation to avoid overlap with neighboring atoms in the lattice are not uniquely defined and different DFT codes use slightly different procedures. For this reason, the PDOS should be considered as a qualitative, rather than a quantitative tool. Nevertheless, the PDOS and more elaborate analysis tools based on it can provide valuable insight into the bonding and band formation in a crystalline solid.

**Figure 10:**
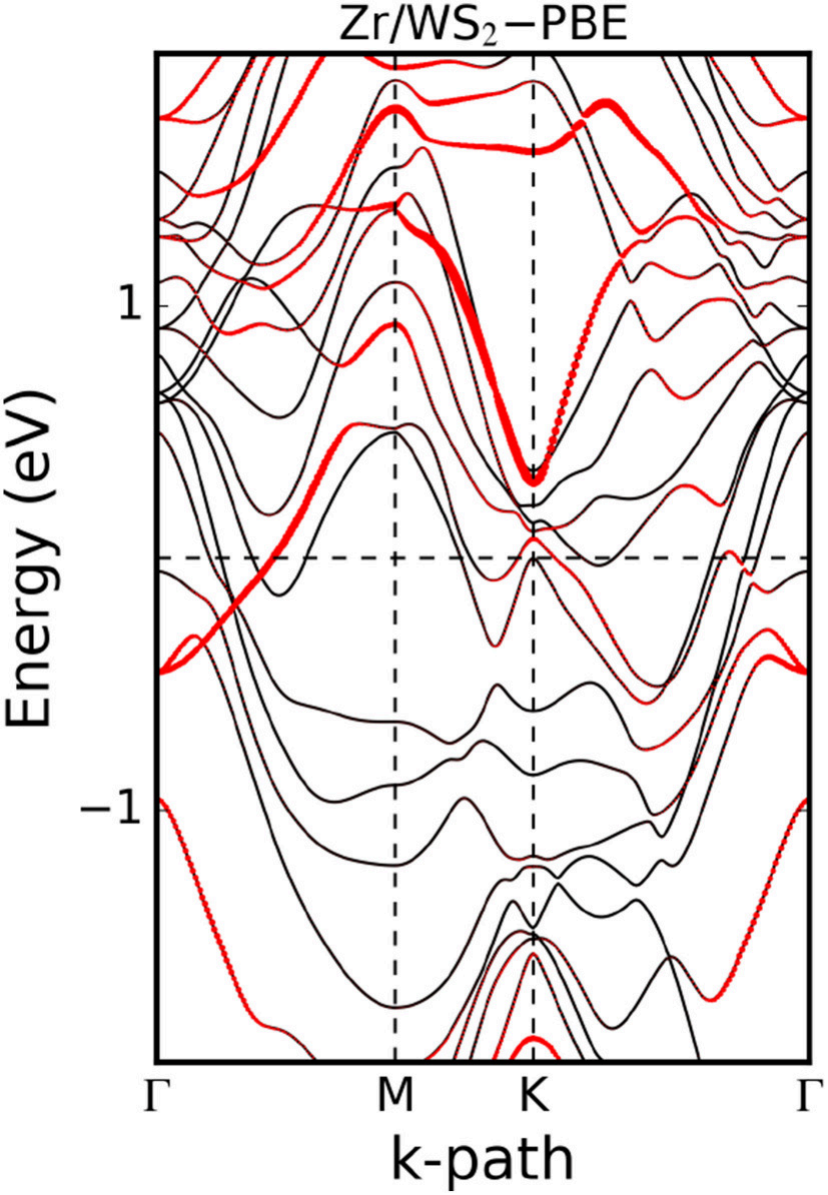
Band structure of the hexagonal unit cell of single-layer WS_2_ contacted by a Zr slab. The size of the red symbols on the bands reflects the W 5d orbital character of the bands. Although the contact as a whole is metallic, one can still see remainders of the valence band and conductance band of the semiconductor WS_2_ in the bands marked by the red symbols. Figure adopted from Kahnouji et al. (2019).

Often it is desirable to provide information about the orbital character of a band when plotting the band structure. For this purpose, the band structure plot is superimposed by the information stemming from the PDOS. For example, a color scale is used to indicate the contribution of a certain orbital to a band, or symbols with sizes proportional to the admixture of a certain orbital are plotted on top of the line plot of the band structure. This is illustrated in Figure 10 for the example of a zirconium contact on the two-dimensional semiconductor WS_2_ (Kahnouji et al., 2019). The band structure and the PDOS have been calculated with the FHI-aims code (Blum et al., 2009).

In practice, the calculation of the various densities of states defined above, as well as of the Fermi surface, is carried out in a post-processing step as a non-self-consistent DFT calculation using the charge density from a previously performed self-consistent (with a Monkhorst-Pack grid) calculation as input. The post-processing can use a finer grid, plus additionally improved sampling via the Blöchl-corrected tetrahedron method (Blöchl et al., 1994).

## 5. Calculations Beyond Total Energy Requiring a Very High Number of k-points

Some semiconductors (e.g., GaAs, GaN, or ZnO) possess sharp minima (small effective masses) of their conduction bands. For obtaining an accurate density of states in the conduction band, a very dense sampling of **k**-space at and around the Γ point is required. Fortunately, the unoccupied conduction band states do not contribute to the total energy; thus, this issue does not affect total energies and stabilities, but shows up when plotting the DOS or PDOS. If this is needed, it is recommendable to interpolate the energy eigenvalues on some already rather dense mesh by analytical functions in the vicinity of Γ. Subsequently, one can evaluate these analytical functions on as many **k**-points as required to obtain the smooth DOS.

More generally, any flat parts in the band structure of low-dimensional systems show up as van Hove singularities (van Hove, 1953) in the density of states. If a band behaves as $\varepsilon_{i}\left(\text{k}\right)\sim {\left(\text{k}-{\text{k}}_{0}\right)}^{2}$ (typically encountered around a high-symmetry point **k**_0_), the density of states *g*(ε) shows a discontinuity (jump) in two dimensions, and even diverges like ~ ε^−1/2^ in a one-dimensional system. If such a behavior is important for the physical effects one wishes to study (e.g., for thermoelectric properties, the skewness of *g*(ε) near the Fermi energy is important), knowledge of the position of van Hove singularities, and a very dense sampling of **k**-space at the corresponding points **k**_0_ are required. Moreover, the relevance of van Hove singularities in superconductivity of metals with high transition temperature is well known.

Problems in magnetism regularly involve an energy scale that is small on the scale of typical electronic energies. Tiny total energy differences are important for magnetic properties of materials. Therefore, highly converged calculations with many **k**-points are required. One example is the magnetocrystalline anisotropy of a ferromagnet. One wishes to find out which orientation of the magnetization (relative to a coordinate system defined by the crystalline lattice vectors) is the energetically most favorable one. This defines the so-called “easy axis” of magnetization. The energy difference between magnetization along the “easy” axis and another, “hard” axis, is very small: typically μeV for the light ferromagnetic elements (such as Fe, Co, Ni) in a cubic environment, and up to meV for heavier atoms (e.g., rare earth elements) or compound materials where the magnetic atoms sit in a symmetry-reduced environment and their orbitals experience crystal field splitting. The physical origin of the magnetocrystalline anisotropy can be traced back to slight deformations of the Fermi surface when the magnetization vector is rotated from one crystalline axis to another. In order to sample these small changes of the Fermi surface, very fine **k**-point meshes (e.g., a 100 × 100 × 100 mesh) may be required (Razee et al., 1999).

A related difficulty is observed if one wants to study transport properties as a function of magnetization, e.g., in case of the anisotropic magneto-resistance (Popescu and Kratzer, 2013), or the so-called giant magnetoresistance (GMR) effect in metallic heterostructures (Heiliger et al., 2008). Here, one is interested in the difference in the conductivity of a magnetic metal if the electrical current flows either parallel or perpendicular to the direction of magnetization (or at any angle with respect to the magnetization vector). Again, the results depend sensitively on fine features of the Fermi surface and their change under a change of magnetization direction.

Adaptive mesh-refinement schemes (AMR) (Bruno and Ginatempo, 1997; Henk, 2001) for Brillouin zone integration provide robust numerical methods which automatically find regions with a high accuracy demand. These regions are sampled with high density, while the other regions are sampled with low density, resulting in considerable savings of computer time as compared to integration methods that rely on equally spaced mesh points. The use of AMR schemes can be traced back to earlier work by Temmerman and Szotek (1987). Since the Brillouin zone integral can be written as three nested integrals over the three dimensions of **k**-space, it is sufficient to illustrate the method for one dimension: The basic idea amounts to concentrating the points at which the integrand is evaluated in those regions where the integrand is large and then perform a quadratic integration. Starting from an equally spaced mesh, in each subsequent step a denser mesh is generated in the critical regions, either by a cascading linear method (halving integration intervals) or by simplex mesh refinement (Henk, 2001). Various indicators may be used to trigger mesh refinement; apart from the absolute size of the integrand these could be sign changes [e.g., in case of the scattering path operator of the Korringa-Kohn-Rostoker (KKR) method (Temmerman and Szotek, 1987)], or the difference between trapezoidal and Simpson rule of integration (Bruno and Ginatempo, 1997).

## 6. Conclusions

With the increasing role of “big data” and high-throughput computations in materials physics and chemistry, it is important to guarantee high accuracy for the first-principles data to be stored in materials databases. For this reason, there is renewed interest in convergence aspects of DFT calculations. There is ample knowledge on the convergence with the sampling of reciprocal space from earlier work with much more limited computational resources. For semiconductors and insulators, this knowledge about special **k**-points can be used to carry out the calculations in the most economic way. For metals and alloys, the accurate sampling of the Fermi surface is still an issue and requires the use of dense **k**-point meshes to ensure a convergence of the total energy per atom to better than 1 meV. Even for simple metals, there is astounding diversity in the shapes of their Fermi surfaces. Thus, there is presently no general recipe available for choosing **k**-points that circumvents a dense sampling of the Brillouin zone. Moreover, for high sampling density, the exact location of each **k**-point, and thus the knowledge about special **k**-point sets, becomes less relevant. If the goal of the calculations is the total energy and the relaxed atomic structure, the most efficient way to deal with the Fermi surface is some broadening of the Fermi-Dirac distribution function. The broadening must be chosen such that a sufficiently large fraction of all occupation numbers shows fractional occupation. The methods available for extrapolating the total energy to the limit of zero broadening work well also with a very high number of **k**-points. The forces on the atoms deserve special attention, since they are calculated as the derivative of the electronic free energy (rather than the total energy), but for the test cases investigated this did not cause problems as long as the Methfessel-Paxton scheme with a broadening parameter in the range of 0.1–0.2 eV and a sufficiently dense **k**-point mesh is used. For post-processing with analysis tools and for quantities beyond the total energy (e.g., for transport properties), integration schemes that go beyond the standard trapezoidal rule, e.g., quadratic interpolation or adaptive **k**-point meshes, turn out to be useful and offer an area for future research.

## Data Availability

The pyiron notebook and the datasets generated for this study will be made available on request to the corresponding author.

## Author Contributions

All authors listed have made a substantial, direct and intellectual contribution to the work, and approved it for publication.

### Conflict of Interest Statement

The authors declare that the research was conducted in the absence of any commercial or financial relationships that could be construed as a potential conflict of interest.

## Footnotes

^1^The points in the first Brillouin zone are closer to the origin than to any other reciprocal lattice point. Due to the periodicity of the factor *e*^*i***kR**^ in Equation (6), **k** points outside this range do not provide any new information.

^2^One applies all symmetry operations in the point group, including subsequent action of several elementary rotations or reflections, to all k-points in the irreducible wedge.

^3^The values of ε_*i*_(**k**_*j*_) at the four apices of the tetrahedron allows us to define a plane intersecting the tetrahedron that is a local (tangent plane) approximation to the global function ε_*i*_(**k**). However, this method can fail when two bands cross inside the tetrahedron. Fortunately, this will happen only in a small fraction of all tetrahedra and the error associated with it only contributes in higher order to the total discretization error.

^4^Note that the VASP code (Kresse and Furthmüller, 1996b) uses σ = 0.2eV as default.

^5^There are alternative geometrical models for a surface, e.g., as a cluster of atoms with one planar face representing the surface and a 'rounded' back side, but the uncontrolled influence of this back side calls for additional measures, e.g., embedding strategies, to obtain converged results, which makes the cluster approach rather complicated if reliable quantitative results should be obtained.

^6^For the use of the supercell method in the calculations of defects, see the review by Spitaler and Estreicher (2018) in the same issue.
